# Peritonsillar abscess subsequently complicated by Ludwig's angina

**DOI:** 10.1002/jgf2.451

**Published:** 2021-05-07

**Authors:** Naoki Matsuura

**Affiliations:** ^1^ Department of Internal Medicine Koga General Hospital Miyazaki Japan

**Keywords:** infectious diseases, internal medicine

## Abstract

The images of this article are clinical pictures of peritonsillar abscess subsequently complicated by Ludwig's angina of a 68‐year‐old man.

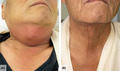

A 68‐year‐old man with ulcerative colitis visited the otolaryngology clinic with a 2‐day history of sore throat and trismus and was diagnosed with a peritonsillar abscess that necessitated needle aspiration. The patient refused the intervention and was administered oral and intravenous antibiotics; however, he subsequently developed swelling and pain in the right side of the neck and treated with frequent doses of oral nonsteroidal anti‐inflammatory drug (NSAID). Five days later, the patient was referred to the emergency department with severe acute abdominal pain, without respiratory distress or dysphonia. Physical examination revealed submandibular swelling and redness (Figure 1A), but showed no dental caries or gingivitis lesions. Contrast‐enhanced computed tomography showed a peritonsillar abscess extending into the submandibular space (Figure 2) and extraluminal free air adjacent to the duodenal bulb. The patient was diagnosed with peritonsillar abscess complicated by Ludwig's angina and duodenal perforation. Following emergency surgical repair of the duodenal perforation, we performed submandibular incision and drainage, and the infection resolved completely after a 6‐week course of intravenous and oral antibiotics (Figure 1B). Twenty‐three days after the admission, we performed a gastroduodenal endoscopy and found a healing ulcer in the anterior wall of the duodenal bulb. Because no clinical or radiological evidence of an association between the duodenal perforation and deep‐neck infections was found, I attributed the duodenal perforation to a NSAID‐induced peptic ulcer.

**FIGURE 1:**
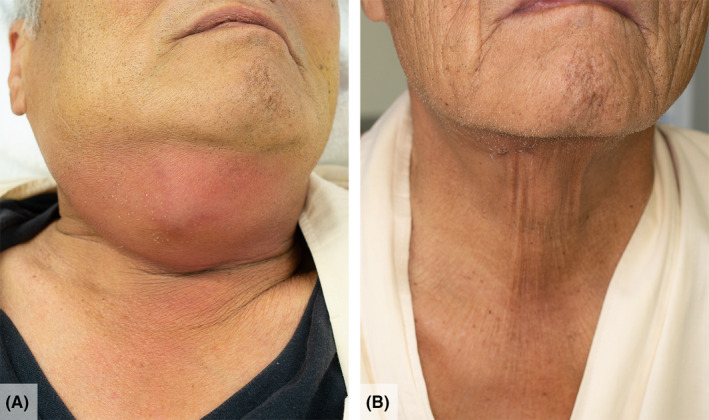
External appearance of the neck. (A) Swelling of the neck on admission; (B) the neck on day 23 of treatment after the swelling resolved

**FIGURE 2:**
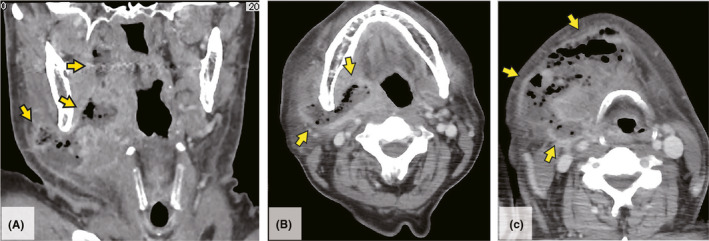
Contrast‐enhanced computed tomography of the neck. (A) Coronal view; (B) axial view at the level of the mandible; (C) axial view at the level of the hyoid bone showing rim‐enhancing fluid and gas collection (yellow arrows)

Peritonsillar abscess is one of the commonest deep‐neck infections and is associated with life‐threatening complications such as descending mediastinitis or parapharyngeal and retropharyngeal abscesses.^1^ Deep‐neck spaces generally play an important role in the spread of deep‐neck infections,usually, peritonsillar abscesses spread posteriorly into the contiguous deep‐neck spaces via the parapharyngeal space, although extension into the anterolateral or inferior deep‐neck spaces has been reported sometimes.^1^, ^2^ The submandibular space is superior and adjacent to the parapharyngeal space, Ludwig's angina, a gangrenous infection of the submandibular space, is usually odontogenic, although peritonsillar abscess could be a possible cause.^3^

## CONFLICT OF INTEREST

The authors have stated explicitly that there are no conflicts of interest in connection with this article.
